# Elaboration of a Radiomics Strategy for the Prediction of the Re-positive Cases in the Discharged Patients With COVID-19

**DOI:** 10.3389/fmed.2021.730441

**Published:** 2021-09-16

**Authors:** Xiao-Hui Wang, Xiaopan Xu, Zhi Ao, Jun Duan, Xiaoli Han, Xing Tang, Yu-Fei Fu, Xu-Sha Wu, Xue Wang, Linxiao Zhu, Wenbing Zeng, Shuliang Guo

**Affiliations:** ^1^Department of Pulmonary and Critical Care Medicine, The First Affiliated Hospital of Chongqing Medical University, Chongqing, China; ^2^School of Biomedical Engineering, Air Force Medical University, Xi'an, China; ^3^Department of Radiology, Xijing Hospital, Air Force Medical University, Xi'an, China; ^4^Department of Radiology, Chongqing University Three Gorges Hospital, Chongqing, China

**Keywords:** COVID-19, re-positive cases, computed tomography, radiomics, prediction

## Abstract

**Objective:** A considerable part of COVID-19 patients were found to be re-positive in the SARS-CoV-2 RT-PCR test after discharge. Early prediction of re-positive COVID-19 cases is of critical importance in determining the isolation period and developing clinical protocols.

**Materials and Methods:** Ninety-one patients discharged from Wanzhou Three Gorges Central Hospital, Chongqing, China, from February 10, 2020 to March 3, 2020 were administered nasopharyngeal swab SARS-CoV-2 tests within 12–14 days, and 50 eligible patients (32 male and 18 female) with completed data were enrolled. Average age was 48 ± 11.5 years. All patients underwent non-enhanced chest CT on admission. A total of 568 radiomics features were extracted from the CT images, and 17 clinical factors were collected based on the medical record. Student's *t*-test and support vector machine–based recursive feature elimination (SVM-RFE) method were used to determine an optimal subset of features for the discriminative model development.

**Results:** After Student's *t*-test, 62 radiomics features showed significant inter-group differences (*p* < 0.05) between the re-positive and negative cases, and none of the clinical features showed significant differences. These significant features were further selected by SVM-RFE algorithm, and a more compact feature subset containing only two radiomics features was finally determined, achieving the best predictive performance with the accuracy and area under the curve of 72.6% and 0.773 for the identification of the re-positive case.

**Conclusion:** The proposed radiomics method has preliminarily shown potential in identifying the re-positive cases among the recovered COVID-19 patients after discharge. More strategies are to be integrated into the current pipeline to improve its precision, and a larger database with multi-clinical enrollment is required to extensively verify its performance.

## Introduction

Since the outbreak of COVID-19 in December 2019, infection by severe acute respiratory syndrome coronavirus 2 (SARS-CoV-2) has led to an increasing number of confirmed patients and deaths all over the world, with an estimated mortality of 4.1% (https://www.who.int/emergencies/diseases/novel-coronavirus-2019). Many patients have recovered and were discharged to the designed place for isolation. In February 2020, Lan et al. first reported that four patients who met the criteria for hospital discharge had positive real-time reverse transcriptase-polymerase chain reaction (RT-PCR) of SARS-CoV-2 test (1). With the increasing number of recovered patients discharged from the hospital by regular follow-up, more and more patients with COVID-19 were found to have re-positive RT-PCR test after discharge (2–5). The proportion of re-positive cases among the discharged patients with COVID-19 varies from 10.6 to 21.4% (4, 6, 7). Reasons for re-positive COVID-19 patients after discharge from the hospital may include the biological characteristics of SARS-CoV-2, clinical status of patients, underlying medical conditions, impact of drug therapy and other treatments, sampling and detection, and re-infection (8). Since re-positive COVID-19 patients after discharge may cause serious consequences as a source of infection (9), early prediction of the re-positive COVID-19 cases among the discharged patients is critically significant to determine the isolation period and develop clinical protocols.

A study reported that there is no significant difference in age between re-positive patients and patients in the control group (6). However, another study reported that the risk of re-positive test after discharge is more than six times higher in persons aged ≥60 years (10). So, there is controversy about risk factors of re-positive patients. Although previous studies have tried to predict the re-positive cases using the clinical features like age, underlying disease, CD4^+^ T lymphocytes, inflammatory indicators, drugs, and duration of treatment (11, 12), the predictive effect is far less than satisfactory (13). Moreover, artificial intelligence and radiomics strategy have been used to fight against COVID-19, including classification of COVID-19 from non-COVID-19 or other pneumonia, severity assessment, and follow-up of COVID-19 (14–16). Until now, as far as we are concerned, no researches have investigated the feasibility and performance of the radiomics strategy for the discrimination of the re-positive cases among the recovered COVID-19 patients after discharge. The purpose of this study was to explore a CT-based radiomics strategy to predict the re-positive case in the test of recovered patients with COVID-19.

## Materials and Methods

### Patients

A total of 91 patients discharged from Wanzhou Three Gorges Central Hospital, Chongqing, China, from February 10, 2020 to March 3, 2020, who followed strict self-isolation or designated isolation, were administered nasopharyngeal swab SARS-CoV-2 test 12–14 days after discharge, and 50 eligible patients with completed data were enrolled [Appendix Figure E1 (online)]. The patients with positive result were defined as “re-positive” group, and the others were “negative” group. All the recovered patients met the discharge criteria according to the *Diagnosis and Treatment of 2019-nCoV Pneumonia in China* (5th edition) (http://www.nhc.gov.cn/): (1) normal temperature for more than 3 days, (2) significant improvement of respiratory symptoms, (3) significant absorption of acute exudative lesions on chest radiograph, and (4) two consecutively negative results by RT-PCR assay of nasal and pharyngeal swabs with at least 1-day interval. The definition of severity of COVID-19 (severe vs. non-severe) at the time of admission is in accordance with the American Thoracic Society guidelines for community-acquired pneumonia (17). Ethical approval was obtained for this retrospective analysis, and informed consent was waived.

Among 91 recovered patients, all 50 recovered patients (32 male and 18 female) with completed detailed clinical features, laboratory findings, and chest CT images on admission were enrolled and underwent SARS-CoV-2 assay. Average age was 48 ± 11.5 years. In total, 24 (48%) patients again have a positive SARS-CoV-2 RT-PCR test result of nasopharyngeal swab. Four patients were marked as severe cases, and the rest were non-severe cases. A total of 17 clinical factors were collected from the institutional medical records, including sex, age, severity level, location of the largest lesion, ratio of lesion area in the thoracic cavity, the slice ID of the CT image with the largest lesion, location of all the lesions on this slice, ratio of all the lesions area in the thoracic cavity on this slice, the number of all the lesions on this slice, leukocytes, neutrophils, lymphocytes, lymphocyte ratio, CD4^+^ T lymphocytes, CD8^+^ T lymphocytes, and CD4^+^/CD8^+^ T lymphocytes, to verify their predictive capacity for the discrimination of the re-positive cases among all the discharged patients.

### Image Acquisition

All patients underwent non-enhanced chest CT with a 64-slice spiral CT scanner (SOMATOM Sensation scanner; Siemens Healthineers). The CT scan parameters were as follows: 120 kVp, 150 mA, 1.5 mm collimation, reconstruction matrix of 512 × 512, slice thickness of 1.0 mm, and high spatial resolution algorithm.

### Region of Interest Definition

**Figure 1:**
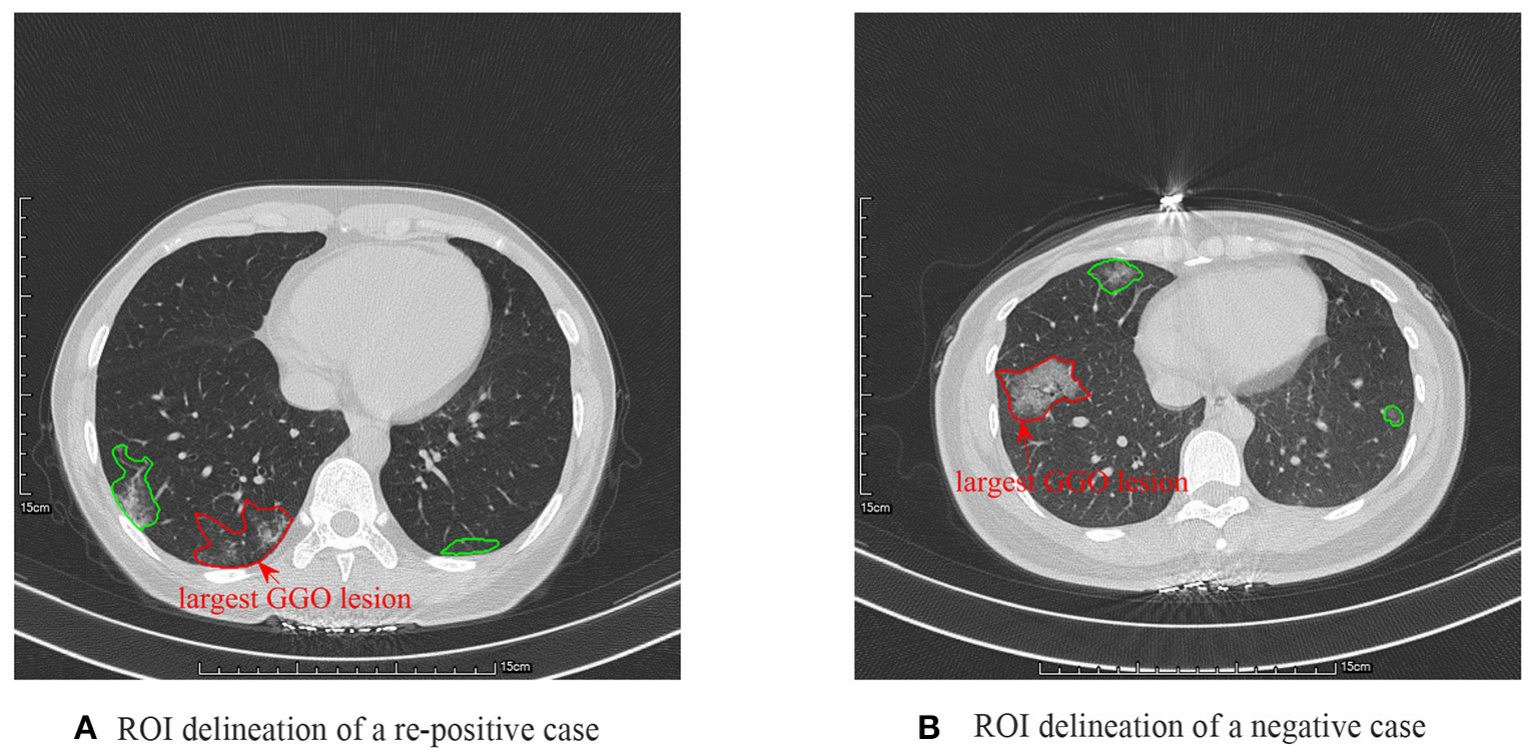
Region of interest delineation results of a re-positive case **(A)** and a negative case **(B)**.

Since the scanning thickness and spacing of the CT images were relatively large, the ground-glass opacity (GGO) lesion size changed dramatically between two neighboring image slices. Therefore, to alleviate the effect of slice thickness and spacing on the analysis, we only selected the CT image slice with the largest GGO lesion region for each patient. And then, the largest GGO lesion region of interest (ROI) was delineated by two radiologists in consensus with a custom-developed package, whose thoracic CT interpretation experience was both more than 9 years, as shown in Figure 1. After that, the other smaller lesions on this image slice were also delineated. The ROIs enclosed by the red curve were the largest GGO lesions of the two cases, respectively, and further used for the radiomics feature extraction, whereas the regions marked by the green curve were the other smaller lesions on this slice, which were used for the computation of some important clinical factors, like the location of all the lesions on this slice, ratio of all the lesions area in the thoracic cavity on this slice, and the number of all the lesions on this slice.

### Radiomics Feature Extraction

After the GGO ROI definition, the next step was radiomics feature extraction. Two groups of radiomics features, including the morphological features and the texture features, were employed to fully describe the lesions on CT images. A total of eight morphological features were extracted from the lesion ROI, including the *major axis length, minor axis length, equivalent diameter, area, eccentricity, orientation, convex area*, and *solidity*. Five categories of texture features, including the histogram features, the second-order texture features like gray-level co-occurrence matrix (GLCM)–based features, the high-order texture features like gray-level run-length matrix (GLRLM)–based features, neighborhood gray-tone difference matrix (NGTDM)–based features, and the gray-level size zone matrix (GLSZM)–based features, were used to comprehensively illustrate the intensity distribution characteristics of the lesions. From each ROI, eight histogram features were extracted.

Considering that the construction of the GLCM, GLRLM, NGTDM, and GLSZM was closely related to the grayscale of the ROI, which would eventually affect the second- and high-order texture features computation, prior to the extraction of these features, a multi-grayscale standardization strategy was used to normalize the grayscale of each ROI into six grayscales widely used in the previous studies (18–20), including 8, 16, 32, 64, 128, and 256. Therefore, for each normalized grayscale, 39 GLCM-based features, 33 GLRLM-based features, 5 NGTDM-based features (21), and 15 GLSZM-based features (22) were calculated from each ROI, and a total of 552 features of these four categories were finally calculated.

After the radiomics feature extraction, eight morphological features and 560 texture features (8 histogram features and 552 second- and high-order texture features) constituted the entire radiomics feature set for the quantitatively describing the geometrical appearance and the local, regional, and global intensity distribution characteristics of the lesion ROI. The detailed information of all these radiomics features is organized in Appendix Table E1 (online).

### Feature Selection

To select the optimal features significantly reflecting the differences between the re-positive and negative cases, a two-step feature selection strategy was employed. First, all the clinical factors and the radiomics features were statistically analyzed using Student's *t*-test to select the features with significant differences (*p* < 0.05) between the re-positive and negative cases. Then, the support vector machine (SVM)–based recursive feature elimination (RFE) algorithm was used for the determination of the optimal features. Detailed description on SVM-RFE has been summarized in Xu et al. (23, 24).

### Development and Validation of the Prediction Model

After feature selection, the prediction models were developed by using the optimal radiomics features, the optimal clinical factors, and both the optimal radiomics and clinical features, and their performance for the discrimination of the re-positive cases was then compared with the quantitative metrics of sensitivity, specificity, accuracy, and the area under the curve (AUC) of receiver operating characteristic. Prior to classification, each feature were normalized to [−1, 1]. Labels of the re-positive cases were set as “+1,” and that of negative cases were set as “−1.” Grid search method was performed in the model training process to select the optimal parameters for the classifier. Considering such a limited database only containing 50 subjects (24 of them were re-positive cases), randomly dividing the database into training and validation cohorts would induce insufficient training and performance validation. Therefore, a 3-fold cross-validation (CV) strategy was used to fully use each of the dataset for model training and validation, and the average results after the 100-round classifications were obtained as the overall performance.

### Ethics Statement

This study was approved by the Medical Ethical Committee of the First Affiliated Hospital of Chongqing Medical University (approval number 20200601). Due to the special reasons of the epidemic, the patients' informed consent was not obtained.

## Results

### Clinical Characteristics

Compared with the control group, the re-positive cases had no significant differences in age, gender, severity of disease, leukocytes, neutrophils, neutrophil ratio, lymphocytes, lymphocyte ratio, CD4^+^ T lymphocytes, CD8^+^ T lymphocytes, CD4^+^/CD8^+^, location of the largest lesion on the cross-sectional slice of the thoracic cavity, area ratio of the largest lesion on the cross-sectional slice of the thoracic cavity, quantified distribution of all the lesions on this slice, area ratio of all the lesions on this slice, the amount of lesions on this slice, and the ID of this slice among the entire CT data. The strategy we adopted to quantify distribution of all the lesions on the same cross-sectional slice is described in the Appendix (online). The demographics and clinical factors of the patients are listed in Table 1.

**Table 1:** Baseline demographics and clinical characteristics of the patients involved in this research.

**Characteristics**	**Re-positive cases (*n* = 24)**	**Negative cases (*n* = 26)**	***p*-value**
**Age, years**			0.777
Median (range)	47 (30, 79)	47 (27, 68)	
**Sex, no. (%)**			0.333
Male	17/24 (71%)	15/26 (58%)	
Female	7/24 (29%)	11/26 (42%)	
**Severity, no. (%)**			0.340
Non-severe cases	21/24 (88%)	25/26 (96%)	
Severe cases	3/24 (12%)	1/26 (4%)	
**Blood routine**			
Leukocytes (× 10^9^/L, normal range 3.5–9.5)	5 (2.7, 8.5)	5.1 (2.1, 10.0)	0.813
Neutrophils (× 10^9^/L, normal range 1.8–6.3)	3.4 (1.3, 7.6)	3.5 (1.1, 8.2)	0.884
Neutrophil ratio (%, normal range 40–75)	66.3 (38.5, 89.0)	67.4 (37.7, 84.6)	0.776
Lymphocytes (× 10^9^/L, normal range 1.1–3.2)	1.2 (0.4, 2.3)	1.1 (0.6, 2.0)	0.880
Lymphocyte ratio (%, normal range 20–50)	23.5 (7.4, 42.0)	24.4 (10.6, 51.3)	0.716
**Lymphocyte classification**			
CD4^+^ T lymphocytes (/μl, normal range 410–1,590)	378 (132–862)	403 (203–767)	0.639
CD8^+^ T lymphocytes (/μl, normal range 190–1,140)	286 (74–621)	269 (129–602)	0.667
CD4^+^/CD8^+^ T lymphocytes (normal range 0.7–2.87)	1.5 (0.59–3.71)	1.6 (0.54–3.1)	0.834
**Location of the largest lesion on the cross-section, no. (%)**			
Upper left lobe	10/24 (42%)	7/26 (27%)	0.272
Lower left lobe	15/24 (63%)	15/26 (58%)	0.729
Upper right lobe	0/24 (0%)	2/26 (8%)	0.491
Middle right lobe	7/24 (29%)	6/26 (23%)	0.435
Lower right lobe	16/24 (62%)	20/26 (77%)	0.420
**Area ratio of the largest lesion on the cross-section, %**			
Median (range)	4.61 (0.17, 67.40)	6.93 (1.09, 33.67)	0.764
**Quantified distribution of all lesions on the same cross-sectional slice, no. (%)**			
Median (range)	8 (3,8)	10.5 (1,27)	0.779
**Area ratio of all lesions on the same cross-sectional slice, %**			
Median (range)	8.47 (0.17, 100)	9.85 (1.58, 63.22)	0.819
**Number of all lesions on the same cross-sectional slice, no**.			
Median (range)	2 (1,11)	3 (1,8)	0.638
**Slice ID of the largest lesion area among the entire CT data**			
Median (range)	437 (60, 573)	417 (32, 614)	0.973

### Results of the Optimal Features Selection

After Student's *t*-test, 62 radiomics features showed significant inter-group differences (*p* < 0.05) between the re-positive cases and the negative cases, including one morphological feature (*eccentricity*), two histogram features (*entropy, uniformity*), and 59 of the second- and high-order texture features. Detailed information of these features is listed in Appendix Table E2 (online). Of the 17 clinical factors, none of them have shown significant inter-group differences between these two groups.

### Performance of the Optimal Features Selected From the 62 Significant Radiomics Features

**Figure 2:**
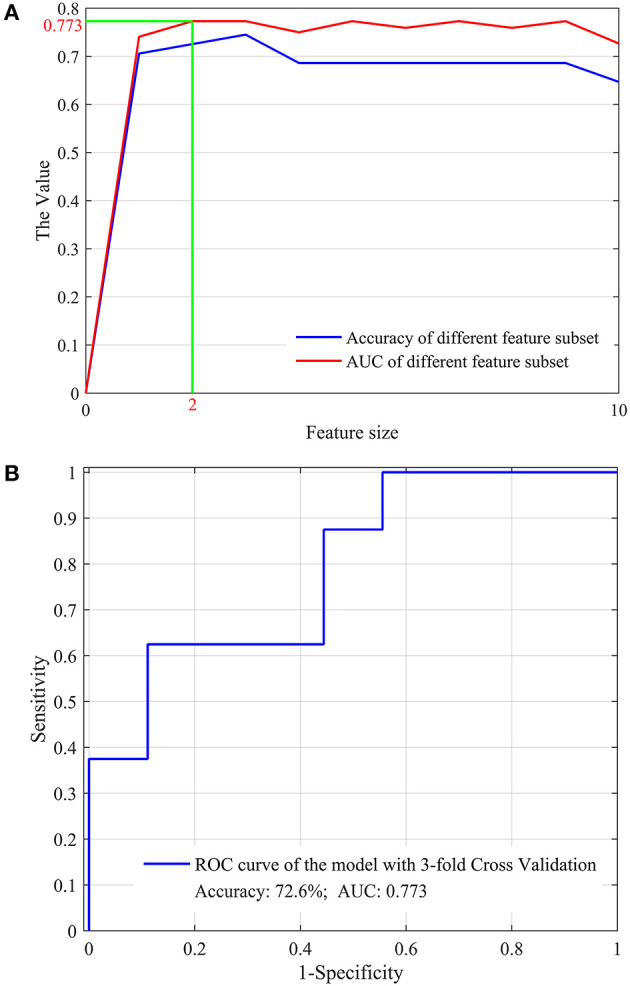
The optimal feature subset selected from the significant radiomics features **(A)** and the overall performance **(B)** for the prediction of the re-positive cases.

Using the 62 significant radiomics features with SVM-RFE method, we further obtained a more compact feature subset with only two radiomics features determined, which achieved the best performance for the prediction of the re-positive cases, as shown in Figure 2A. The two features were GLRLM-11-16GL and GLRLM-11-8GL, which represented the 11th feature of the GLRLM feature category, namely, the long run high gray-level emphasis (LRHGE), extracted from the ROIs with the intensity grayscale normalized to 16 and 8, respectively. The prediction model was then developed using these two features and an SVM classifier, and its overall performance with 3-fold CV and 100-round classification was fair and acceptable, as shown in Figure 2B.

### Performance of the Optimal Features Selected From the 17 Clinical Factors

**Figure 3:**
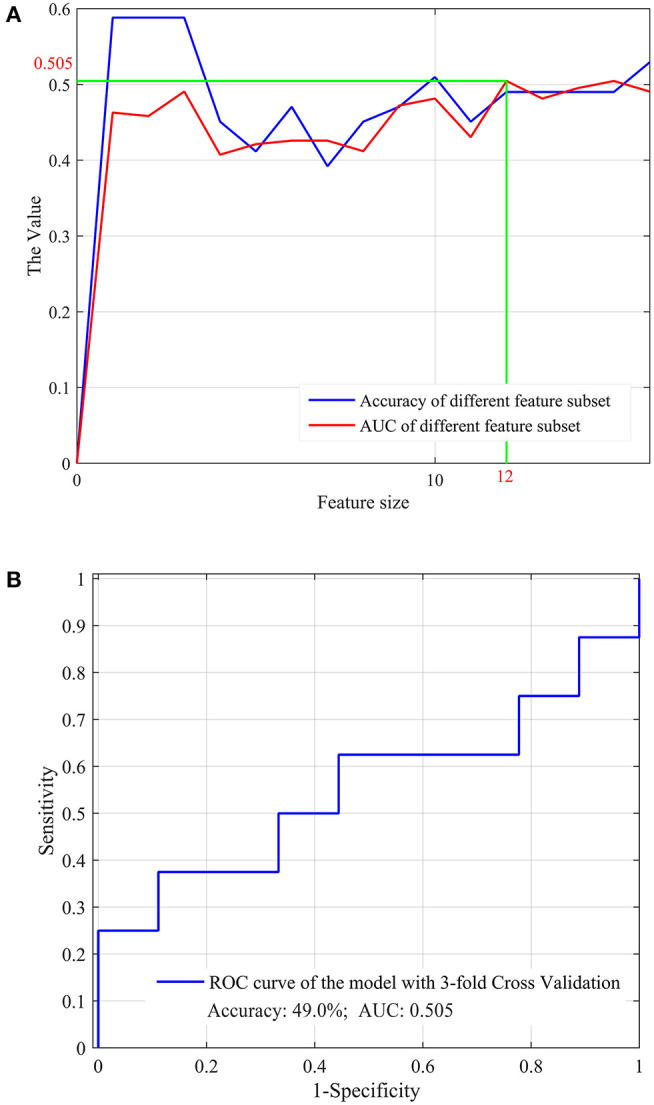
The optimal feature subset selected from the clinical factors **(A)** and the overall performance **(B)** for the prediction of the re-positive cases.

Although no clinical factor showed significant inter-group differences between the re-positive cases and the negative ones, these factors were still used for the optimal feature selection and prediction model development. The results showed that 12 factors were determined as the optimal factors, as shown in Figure 3A, and achieved the best prediction performance, with the accuracy and AUC of 49.0 and 0.505, respectively, as shown in Figure 3B.

### Performance of the Optimal Features Selected From the Radiomics Features and the Clinical Factors

**Figure 4:**
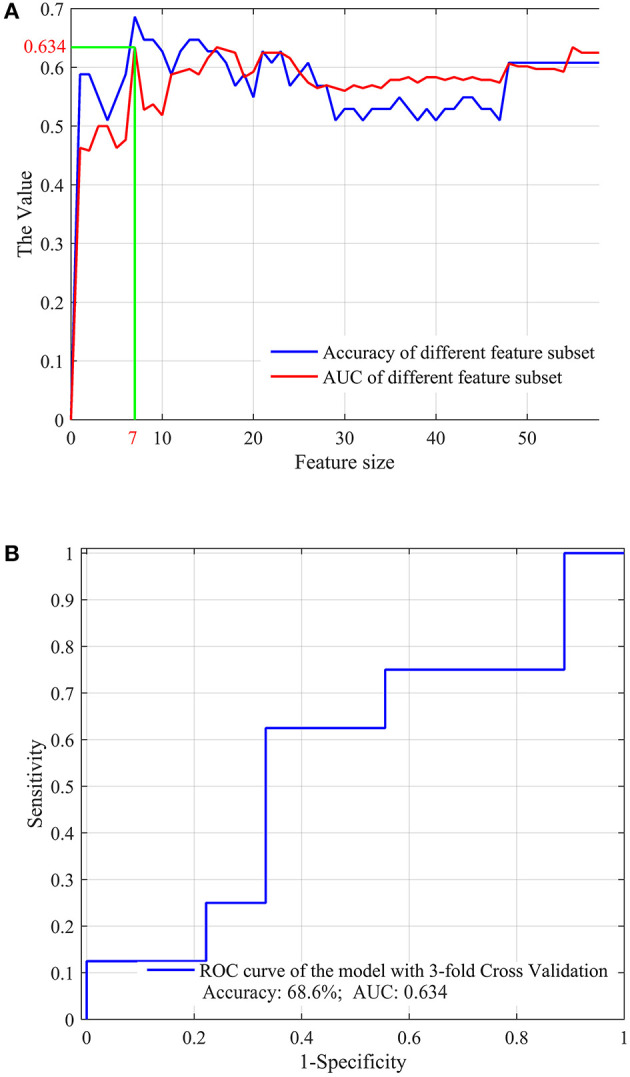
The optimal feature subset selected from the significant radiomics features and the clinical factors **(A)** and the overall performance **(B)** for the prediction of the re-positive cases.

We further selected the optimal features from the significant radiomics features and all the clinical factors, aiming to evaluate the performance of combining the radiomics features with the clinical factors for the prediction task. The optimal feature determination process is shown in Figure 4A, and the prediction performance using these optimal features and an SVM classifier is shown in Figure 4B.

Table 2 shows the performance comparison of the prediction models developed by using the optimal radiomics features, the optimal clinical factors, and both the optimal radiomics and clinical features, and their performance for the discrimination of the re-positive cases was then compared, which indicates the superiority of the radiomics features for the re-positive case prediction.

**Table 2:** Discriminative capability of different nomogram models in MI prediction.

**Prediction model**	**Feature size**	**Sensitivity (%)**	**Specificity (%)**	**Accuracy (%)**	**AUC**
Optimal clinical factors	12	41.7	55.6	49.0	0.505
**Optimal radiomic features**	2	70.8	74.1	72.6	0.773
Combined	7	58.3	77.8	68.6	0.634

## Discussion

Although previous studies have tried to predict the re-positive RT-PCR test of COVID-19 through clinical features, the predictive effect is not very satisfactory (13). It remains to be determined whether the clinical features are related to the re-positivity of COVID-19 after discharge. Yan et al. reported that older age and the lack of lopinavir/ritonavir treatment were independently associated with prolonged SARS-CoV-2 RNA shedding in patients with COVID-19 (25). In another study, it was also confirmed that the risk of re-positive test after discharge is more than six times higher in persons aged 60 years and above (10), whereas Xiao et al. reported that there is no significant difference in age between re-positive patients and patients in the control group (6). In this study, our data showed that clinical characteristics and laboratory indicators were not effective in predicting the possibility of re-positivity in patients with COVID-19, including age, sex, severity of disease, leukocytes, neutrophils, neutrophil ratio, lymphocytes, lymphocyte ratio, CD4^+^ T lymphocytes, CD8^+^ T lymphocytes, CD4^+^/CD8^+^, location of the largest lesion on the cross-sectional slice of the thoracic cavity, area ratio of the largest lesion on the cross-sectional slice of the thoracic cavity, quantified distribution of all the lesions on this slice, area ratio of all the lesions on this slice, the amount of lesions on this slice, and the ID of this slice among the entire CT data. This reflects the need to assess not only clinical symptoms but also radiological features and assessing whether a patient can be discharged from hospital.

Currently, the diagnosis, isolation, and discharge mainly depend on RT-PCR of SARS-CoV-2. Ai et al. reported that the sensitivity of chest CT based on positive RT-PCR results in detecting COVID-19 was 97% (26). In addition, chest CT findings can be more early and sensitive than RT-PCR in diagnosis of COVID-19. Fang et al. showed that the sensitivity of chest CT was greater than that of RT-PCR, namely the detection rate of initial chest CT and RT-PCR and reported a higher detection rate for initial CT (98%) than first RT-PCR (71%) patients (*p* < 0.001) (27). In the research of Long et al., CT sensitivity was 97.2%, whereas the sensitivity of RT-PCR was only 83.3% at initial presentation (28). Ducray et al. reported that the accuracy, sensitivity, and specificity of positive chest CT results respectively reached 88.9, 90.2, and 88%, which are relative to the final RT-PCR test (29). Compared with chest CT, there are many factors affecting the detection process of RT-PCR, so it can be easily restricted by false-negative results.

Chest CT may be considered as a primary tool for the detection of COVID-19 because it is readily performed and obtained. Furthermore, CT can also assess the disease severity and differential diagnosis, and monitor the course of COVID-19 to guide clinical management (30, 31). The greatest severity of lung disease on CT is reported about 10 days and chest CT signs of improvement began at ≥14 days after the onset of initial symptoms (32). As the development of artificial intelligence system, which may be a helpful tool for radiologists, it is possible to improve their work efficiency by identifying COVID-19 from other pneumonia with good accuracy and less time (33). Murphy et al. evaluated the performance of an AI system for detecting COVID-19 pneumonia on chest radiographs, and their results showed that the AI system correctly classified chest X-ray images as COVID-19 pneumonia with an AUC of 0.81 (34). Bai et al. established and evaluated an AI system for differentiating COVID-19 and other pneumonia on chest CT, which demonstrated that their model can improve radiologists' performance better than without it (90 vs. 85% accuracy, 88 vs. 79% sensitivity, 91 vs. 88% specificity) (35). In the current study, clinical characteristics were not effective in predicting the possibility of re-positivity in patients with COVID-19, while our models developed by using the optimal radiomics features showed predictive capability with an accuracy of 72.6%. These results have preliminarily shown potential in identifying the re-positive cases among the recovered COVID-19 patients after discharge.

However, the results of this study should be carefully interpreted due to the following limitations. First of all, the sample size of our study is relatively small and single centered, which might cause certain influence on the generalizability of the predictive model for multicenter applications. Second, a retrospective decision might have a potential impact on these findings. While there was no significant intergroup difference of 17 demographic and clinical characteristics between re-positive and negative patients, given such a limited sample set size, the findings in our study might underestimate the predictive capability of the clinical factors for predicting the re-positive cases. However, the baseline data showed no significant intergroup difference that may highlight the importance and reliability of the results predicted by radiomics features. Other limitations including insufficient non-viral pneumonia controls, without timely and sensitive diagnostic feedback criteria for COVID-19 infection, and imaging diagnosis are non-specific to identify COVID-19 from a variety of viral pneumonia. Multicenter data with subtle differences between scans from different countries, institutions, or CT instruments may better support the generalizability of the findings.

## Data Availability Statement

The original contributions presented in the study are included in the article/Supplementary Material, further inquiries can be directed to the corresponding author/s.

## Ethics Statement

The studies involving human participants were reviewed and approved by Medical Ethical Committee of the First Affiliated Hospital of Chongqing Medical University. Written informed consent for participation was not required for this study in accordance with the national legislation and the institutional requirements.

## Author Contributions

X-HW and SG made substantial contributions to the study concept and design. X-HW was in charge of the article draft. XX participated in drafting of the article and was in charge of image data analysis. XT, Y-FF, and X-SW participated in dealing with images. ZA, XW, WZ, and LZ collected and confirmed data accuracy and images. JD applied for the ethical approval. XH and SG were the clinical experts. WZ was the radiologist in charge of the treatment of the patients. All authors made substantial revisions to the article.

## Funding

This study was supported by: (1) National Natural Science Foundation of China under Grant No. 81901698; (2) Young Eagle Plan of High Ambition Project under grant No. 2020CYJHXXP; (3) Chongqing Education Board “COVID-19's Infection and Prevention” Emergency Scientific Research Project No. KYYJ202006; (4) Chongqing Science and Technology Bureau “COVID-19's Epidemic Emergency Science and Technology Special” the Fourth Batch of Projects No. cstc2020jscx-fyzxX0040; (5) Chongqing Technology Foresight and System Innovation Project No. ctsc2020jsyj-zzysbA0074.

## Conflict of Interest

The authors declare that the research was conducted in the absence of any commercial or financial relationships that could be construed as a potential conflict of interest.

## Publisher's Note

All claims expressed in this article are solely those of the authors and do not necessarily represent those of their affiliated organizations, or those of the publisher, the editors and the reviewers. Any product that may be evaluated in this article, or claim that may be made by its manufacturer, is not guaranteed or endorsed by the publisher.
