# Supplementary material for: Elaboration of a Radiomics Strategy for the Prediction of the Re-positive Cases in the Discharged Patients With COVID-19
Source: Front Med (Lausanne). 2021 Sep 16;8:730441. doi: 10.3389/fmed.2021.730441 (PMC8481365; doi:10.3389/fmed.2021.730441)
Supplement: Supplementary file 1 [file Data_Sheet_1.docx]

**Appendix** **E1: Subjects Enrollment, Radiomics Features Extraction and Feature Selection**

**I. Subject Recruitment and Imaging parameters Description**

The subject recruitment guideline in this study was shown in Figure E1.


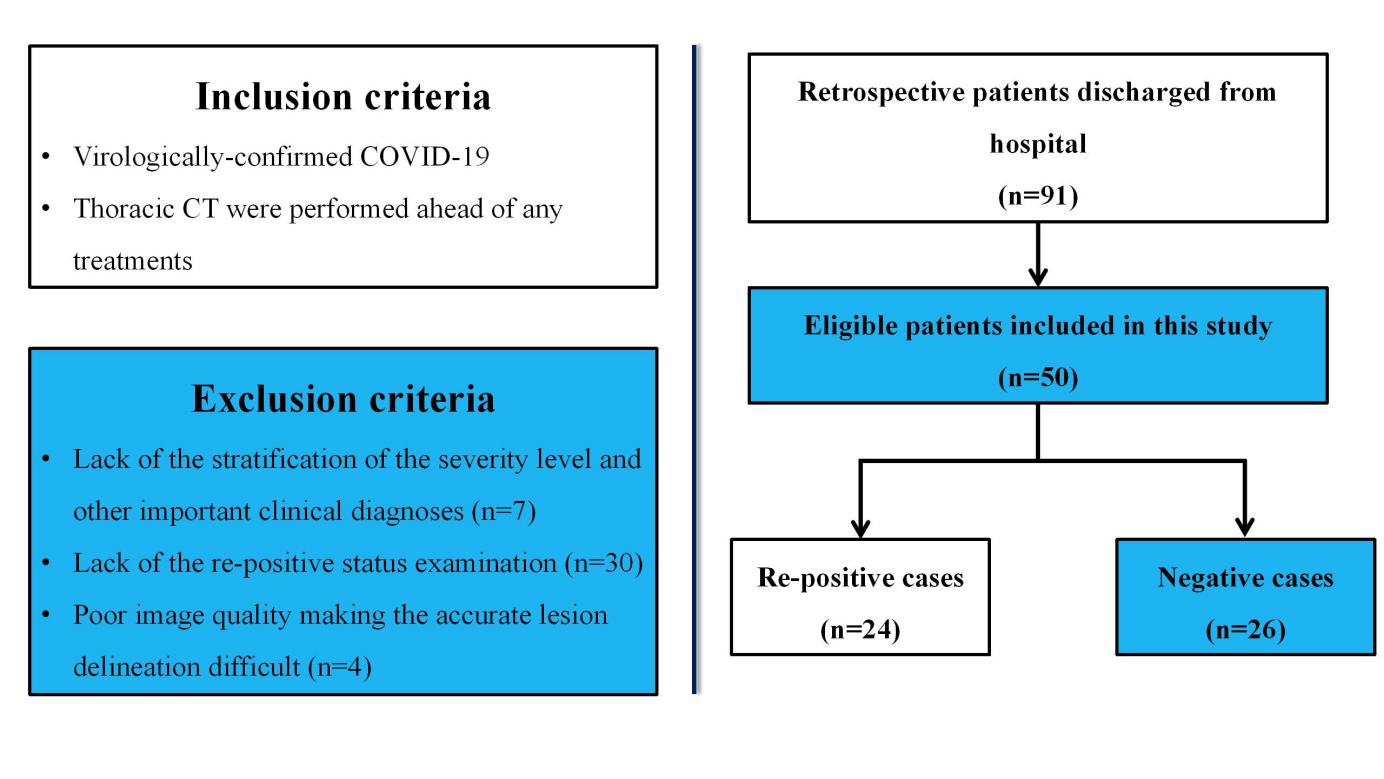


**Figure E1**: The subject recruitment guideline in this study

**II. Radiomics Feature Extraction**

After ROI delineation, the radiomics features, including the morphological features, first-order texture features [[1-5](#_ENREF_1)] (histogram features), second-order texture features[[2-4](#_ENREF_2), [6](#_ENREF_6)] (Haralick features extracted from the co-occurrence matrix, GLCM features hereafter), and high-order texture features [[2](#_ENREF_2), [4](#_ENREF_4), [7-11](#_ENREF_7)] involving features extracted from the run-length matrix (GLRLM), neighborhood gray-tone difference matrix (NGTDM) and gray level size zone matrix (GLSZM), i.e., GLRLM features, NGTDM features and GLSZM features hereafter, were calculated from each ROI set to fully characterize the tissue distribution patterns within the lesion region. Specifically, prior to second-order and high-order feature extraction, the image intensity was discretized and normalized to five standard grayscales (8, 16, 32, 64, 128 and 256). Then, these four texture feature groups were extracted from the ROIs using each normalized grayscale.

***Morphological features***

A total of eight extensively-used morphological features were extracted from each lesion ROI, including the *Major Axis Length*, *Minor Axis Length*, *Equivalent Diameter*, *Area*, *Eccentricity*, *Orientation*, *Convex Area*, *Solidity*. These features are listed in Table E1.

***Histogram features***

Eight widely used histogram-based features [[1](#_ENREF_1), [2](#_ENREF_2), [4](#_ENREF_4)], including the *mean*, entropy, *uniformity*, *standard deviation*, *smoothness*, *skewness*, *third-order moment and kurtosis*, were applied to fully describe the global texture patterns within the lesion. These features are listed in Table E1.

***GLCM features***

GLCM features were proposed by Haralick et al.[[6](#_ENREF_6)]. They describe the pixel-paired distribution within images, which may reflect the local heterogeneity of tissues [[4](#_ENREF_4), [6](#_ENREF_6), [12](#_ENREF_12)]. For each ROI, four GLCMs were computed along four principal directions (0°, 45°, 90°, and 135°) with a distance between two pixels set as “1”. For each GLCM, Haralick et al. proposed 13 measures to characterize the tissue patterns [[1](#_ENREF_1), [6](#_ENREF_6), [12](#_ENREF_12)]. Therefore, for each Haralick measure, four values were eventually obtained from the four GLCMs. To keep the features rotationally invariant, for each measure, the average, range (difference between the maximum and minimum values) and standard deviation of its four values were calculated [[6](#_ENREF_6), [12](#_ENREF_12)]. Eventually, a total of 39 GLCM features were obtained (see Table E1).

***GLRLM features***

The run-length is used to evaluate the contiguous gray level along a predefined direction. Therefore, the GLRLM features are capable of describing the regional pixel distribution within the ROI, and can thus reflect the regional heterogeneity of tissues.

For a given picture *Image I*, four GLRLMs can be computed along four principal directions (0°, 45°, 90°, and 135°). In each GLRLM, the matrix element *(m, n)* specifies the number of times that the picture contains a run of length *n* in a given direction, consisting of points having the gray-level *m*. Then, similar to the GLCM features, we calculated the average, range and standard deviation of the 13 measures of the four RLMs, constituting 33 GLRLM features in this group (Table E1).

***NGTDM features***

Textural features corresponding to the visual perception of image texture are highly desirable for pattern recognition and artificial intelligence[[10](#_ENREF_10)]. Neighborhood gray tone difference matrix (NGTDM)-based features were generated by Amadasun and King [[10](#_ENREF_10)], the purpose of which is to quantitatively describe the visual perception properties of image texture and to build a bridge between images and interpretation. The following five metrics are involved in this feature group:

(1) *NGTDM Coarseness*: Coarseness is the most fundamental property of texture, and in a narrow sense, it is used to imply texture [[10](#_ENREF_10)]. If the texture of an image is coarser, it means the primitives or basic patterns of the texture are large, which, in turn, tends to yield a texture with a high degree of local uniformity in intensity [[10](#_ENREF_10)].

(2) *NGTDM Contrast:* An image has a high level of contrast if areas of different gray levels are clearly visible [[10](#_ENREF_10)]. Thus, high contrast means that the intensity difference between neighboring regions is large [[10](#_ENREF_10)] and vice versa.

(3) *NGTDM Busyness:* A busy texture is one in which there are rapid changes in intensity from one pixel to neighboring pixels, which means that the spatial frequency of intensity changes in the region is very high [[10](#_ENREF_10)].

(4) *NGTDM Complexity*: Complexity indicates the visual information content of a texture. A texture is considered complex if the information content is high [[10](#_ENREF_10)].

(5) *NGTDM Strength:* A texture is generally referred to as strong when the primitives of the texture are easily definable and clearly visible [[10](#_ENREF_10)]. All these features are listed in Table E1.

***GLSZM features***

Gray-level size-zone matrix (GLSZM)-based features were generated and described by Thibault et al. [[4](#_ENREF_4), [8](#_ENREF_8), [11](#_ENREF_11)]. A GLSZM describes the number of homogeneous connected areas of a certain size and intensity within the region of interest (ROI) [[4](#_ENREF_4)]. The element *p(i, j)* of the GLSZM represents the number of connected regions of gray level *i* and size *j*. The features derived from the GLSZM, therefore, describe the homogeneous regions within the lesion ROI and can be used to quantitatively assess the lesion heterogeneity in an ROI [[4](#_ENREF_4)]. The feature group contains 15 widely reported metrics (Table E1).

Table E1 Radiomic features extracted from each modality

| Feature category | Feature ID | Description |
| --- | --- | --- |
| Morphological features | M1 | Major Axis Length |
|  | M2 | Minor Axis Length |
|  | M3 | Equivalent Diameter |
|  | M4 | Area |
|  | M5 | Eccentricity |
|  | M6 | Orientation |
|  | M7 | Convex Area |
|  | M8 | Solidity |
| Histogram | H1 | Mean |
|  | H2 | Entropy |
|  | H3 | Uniformity |
|  | H4 | Standard deviation |
|  | H5 | Smoothness |
|  | H6 | Skewness |
|  | H7 | Third order moment |
|  | H8 | kurtosis |
| GLCM features | CM1 | Energy |
|  | CM 2 | Contrast |
|  | CM 3 | Correlation |
|  | CM 4 | Variance |
|  | CM 5 | Inverse difference moment |
|  | CM 6 | Sum average |
|  | CM 7 | Sum variance |
|  | CM 8 | Sum entropy |
|  | CM 9 | Entropy |
|  | CM 10 | Difference variance |
|  | CM 11 | Difference entropy |
|  | CM 12 | Information measures I of correlation |
|  | CM 13 | Information measures II of correlation |
|  | CM 14～26 | Range of the corresponding features listed above |
|  | CM 27～39 | Standard deviation of the corresponding features listed above |
| GLRLM features | RLM1 | Short run emphasis |
|  | RLM 2 | Long run emphasis |
|  | RLM 3 | Gray-level non-uniformity |
|  | RLM 4 | Run length non-uniformity |
|  | RLM 5 | Run percentage |
|  | RLM 6 | Low gray-level run emphasis |
|  | RLM 7 | High gray-level run emphasis |
|  | RLM 8 | Short run low gray-level emphasis |
|  | RLM 9 | Short run high gray-level emphasis |
|  | RLM 10 | Long run low gray-level emphasis |
|  | RLM 11 | Long run high gray-level emphasis |
|  | RLM 12～22 | Range of corresponding features listed above |
|  | RLM 23～33 | Standard deviation of corresponding features listed above |
| NGTDM features | N1 | NGTDM Coarseness |
|  | N2 | NGTDM Contrast |
|  | N3 | NGTDM Busyness |
|  | N4 | NGTDM Complexity |
|  | N5 | NGTDM Strength |
| GLSZM features | SZ1 | Small Area Emphasis (SAE) |
|  | SZ2 | Large Area Emphasis (LAE) |
|  | SZ3 | Low Intensity Emphasis (LIE) |
|  | SZ4 | High Intensity Emphasis (HIE) |
|  | SZ5 | Low Intensity Small Area Emphasis (LISAE) |
|  | SZ6 | High Intensity Small Area Emphasis (HISAE) |
|  | SZ7 | Low Intensity Large Area Emphasis (LILAE) |
|  | SZ8 | High Intensity Large Area Emphasis (HILAE) |
|  | SZ9 | Intensity Nonuniformity (IN) |
|  | SZ10 | Intensity Nonuniformity Normalized (INN) |
|  | SZ11 | Size Zone Nonuniformity (SZN) |
|  | SZ12 | Size Zone Percentage (SZP) |
|  | SZ13 | Intensity Variance (IV) |
|  | SZ14 | Size Zone Variance (SZV) |
|  | SZ15 | Size Zone Entropy (SZE) |

Prior to CM, RLM, NGTDM and GLSZM feature extraction, the grayscale normalization process is required. In this study, six commonly used grayscales, i.e., 8, 16, 32, 64, 128 and 256, were adopted to fully characterize the tissue distribution patterns with the lesion ROI. We allocated a specific feature ID to each feature, featureID-GL, where GL stands for grayscale. For example, the feature “RLM32-16GL” denotes the standard deviation of “Long run low gray-level emphasis” in the RLM feature category calculated from the normalized CT images with the grayscale of 16.

**III. The strategy for quantifying the distribution of all the lesions on the cross-sectional slice**

To quantitatively evaluate the distribution of all the lesions on the same cross-sectional slice that the largest lesion was on, we adopted a quantifying strategy which used five scores (1, 2, 3, 4, 5) to represent the location of each lesion when it was in the upper right lobe, middle right lobe, lower right lobe, upper left lobe and lower left lobe, respectively. With all the scores of each lesion accumulated, the final score would be the quantified result of the distribution of all the lesions on the cross-sectional slice.

**IV. Feature selection using the Student’s *t*-test analysis**

After the Student’s *t*-test, 62 radiomics features showed significant inter-group differences (*p* < 0.05) between the re-positive cases and the negative cases, including one morphological feature (*Eccentricity*), two histogram features (*Entropy*, *Uniformity*), and 59 of the second- and high-order texture features. Detail information of these features was listed in Table E2. Of the 16 clinical factors, none of them shown significant inter-group differences between these two groups.

Table E2 Radiomic features with significant inter-group differences (*p* < 0.05)

| Feature category | Total number | Features Selected |
| --- | --- | --- |
| Morphological features | 1 | M5 |
| Histogram | 2 | H2, H3 |
| GLCM features | 35 | CM5-32GL, CM1-64GL, CM3-64GL, CM4-64GL, CM5-64GL, CM6-64GL, CM7-64GL, CM8-64GL, CM9-64GL, CM10-64GL, CM11-64GL, CM1-128GL, CM3-128GL, CM4-128GL, CM5-128GL, CM6-128GL, CM7-128GL, CM8-128GL, CM9-128GL, CM10-128GL, CM11-128GL, CM17-128GL, CM19-128GL, CM20-128GL, CM21-128GL, CM30-128GL, CM32-128GL, CM33-128GL, CM34-128GL, CM3-256GL, CM4-256GL, CM5-256GL, CM6-256GL, CM9-256GL, CM11-256GL |
| GLRLM features | 4 | RLM11-8GL, RLM11-16GL, RLM13-64GL, RLM24-64GL |
| NGTDM features | 3 | N3-8GL, N3-16GL, N1-64GL |
| GLSZM features | 17 | SZ8-8GL, SZ8-16GL, SZ8-32GL, SZ14-32GL, SZ1-64GL, SZ2-64GL, SZ6-64GL, SZ8-64GL, SZ10-64GL, SZ14-64GL, SZ15-64GL, SZ6-128GL, SZ8-128GL, SZ14-128GL, SZ6-256GL, SZ8-256GL, SZ14-256GL |

**V. References**

1. Xu X, Liu Y, Zhang X, Tian Q, Wu Y, Zhang G, et al. Preoperative prediction of muscular invasiveness of bladder cancer with radiomic features on conventional MRI and its high-order derivative maps. Abdominal radiology. 2017; 42: 1896-905.

2. Lambin P, Rios-Velazquez E, Leijenaar R, Carvalho S, van Stiphout RGPM, Granton P, et al. Radiomics: Extracting more information from medical images using advanced feature analysis. European journal of cancer (Oxford, England : 1990). 2012; 48: 441-6.

3. Zhang X, Xu X, Tian Q, Li B, Wu Y, Yang Z, et al. Radiomics assessment of bladder cancer grade using texture features from diffusion-weighted imaging. Journal of magnetic resonance imaging : JMRI. 2017; 46: 1281-8.

4. Lambin P, Leijenaar RTH, Deist TM, Peerlings J, de Jong EEC, van Timmeren J, et al. Radiomics: the bridge between medical imaging and personalized medicine. Nature reviews Clinical oncology. 2017; 14: 749-62.

5. Xu X, Wang H, Du P, Zhang F, Li S, Zhang Z, et al. A predictive nomogram for individualized recurrence stratification of bladder cancer using multiparametric MRI and clinical risk factors. Journal of Magnetic Resonance Imaging. 2019 Apr 13; 0.

6. Haralick RM, Shanmugam K, Dinstein IH. Textural Features for Image Classification. IEEE Transactions on Systems, Man, and Cybernetics. 1973; SMC-3: 610-21.

7. Galloway MM. Texture Analysis Using Gray Level Run Lengths. Computer Graphics and Image Processing. 1975; 4: 172-9

8. Thibault G, Angulo J, Meyer F. Advanced statistical matrices for texture characterization: Application to DNA chromatin and microtubule network classification. IEEE International Conference on Image Processing; 2011. p. 53-6.

9. Thibault G, Angulo J, Meyer F. Advanced statistical matrices for texture characterization: application to cell classification. IEEE Transactions on Biomedical Engineering. 2014; 61: 630-7.

10. Amadasun M, King R. Texural Features Corresponding to Texural Properties. IEEE Transactions on Systems, Man, and Cybernetics. 1989; 19: 1264-74.

11. Thibault G, Fertil B, Navarro C, Pereira S, Levy N, Sequeira J, et al. Texture Indexes and Gray Level Size Zone Matrix Application to Cell Nuclei Classification. In Pattern Recognition and Information Processing (PRIP. 2009.

12. Xu X, Zhang X, Tian Q, Wang H, Cui L-B, Li S, et al. Quantitative Identification of Nonmuscle-Invasive and Muscle-Invasive Bladder Carcinomas: A Multiparametric MRI Radiomics Analysis. Journal of magnetic resonance imaging : JMRI. 2018; 10.1002/jmri.26327.
